# Trauma-related psychological disorders among Palestinian children and adults in Gaza and West Bank, 2005-2008

**DOI:** 10.1186/1752-4458-3-21

**Published:** 2009-09-23

**Authors:** Emmanuelle Espié, Valérie Gaboulaud, Thierry Baubet, German Casas, Yoram Mouchenik, Oliver Yun, Rebecca F Grais, Marie Rose Moro

**Affiliations:** 1 Epicentre, Paris, France; 2 Hôpital Avicenne, Assistance Publique Hôpitaux de Paris, Bobigny, France; 3 Médecins Sans Frontières-France, Paris, France; 4 Université Toulouse Le Mirail, Toulouse, France; 5 Médecins Sans Frontières/Doctors Without Borders, New York, USA; 6 Hôpital Cochin, Maison des adolescents, Université Paris Descartes, AP-HP, France

## Abstract

**Background:**

Trauma from war and violence has led to psychological disorders in individuals living in the Gaza strip and West Bank. Few reports are available on the psychiatric disorders seen in children and adolescents or the treatment of affected populations. This study was conducted in order to describe the occurrence and treatment of psychiatric disorders in the Palestinian populations of the Gaza strip and Nablus district in the West Bank.

**Methods:**

From 2005 to 2008, 1369 patients aged more than 1 year were identified through a local mental health and counseling health network. All were clinically assessed using a semi-structured interview based on the DSM-IV-TR criteria.

**Results:**

Among 1254 patients, 23.2% reported post-traumatic stress disorder [PTSD], 17.3% anxiety disorder (other than PTSD or acute stress disorder), and 15.3% depression. PTSD was more frequently identified in children ≤ 15 years old, while depression was the main symptom observed in adults. Among children ≤ 15 years old, factors significantly associated with PTSD included being witness to murder or physical abuse, receiving threats, and property destruction or loss (p < 0.03). Psychological care, primarily in the form of individual, short-term psychotherapy, was provided to 65.1% of patients, with about 30.6% required psychotropic medication. Duration of therapy sessions was higher for children ≤ 15 years old compared with adults (p = 0.05). Following psychotherapy, 79.0% had improved symptoms, and this improvement was significantly higher in children ≤ 15 years old (82.8%) compared with adults (75.3%; p = 0.001).

**Conclusion:**

These observations suggest that short-term psychotherapy could be an effective treatment for specific psychiatric disorders occurring in vulnerable populations, including children, living in violent conflict zones, such as in Gaza strip and the West Bank.

## Background

The effects of war and violence on the mental health of children and adults are well known and are often expressed through severe and chronic reactive psychological syndromes, including post-traumatic stress disorder (PTSD), anxiety, and behavioral problems [1-4]. The Palestinian population living in the occupied territories has been subjected to continuous violence, such as shooting, bombardment, and physical injuries. As a result of this ongoing crisis, Palestinians, especially women and children, have developed severe psychological distress [5-9]. Although these patients require immediate care, usually mental health and social support, access to health care remains difficult due to settlement policy and creation of enclaves [10,11].

The international medical nongovernmental organization Médecins Sans Frontières/Doctors Without Borders (MSF) has been present in the Palestinian territories since 1999. In addition to emergency-oriented programs, MSF has implemented, in collaboration with local authorities, primary health care programs in different areas of the Occupied Palestinian Territories. Started in November 2000, one of these programs provides adapted medical and psychological care to the population.

Here, we describe the characteristics of psychiatric disorders in Palestinian children and adults among individuals participating in the MSF program in the Gaza strip and Nablus district of the West Bank from 2005 to 2008.

## Methods

All residents of the Gaza strip and West Bank's Nablus district were eligible for free-cost treatment in the MSF program. Patients were identified by an active case finding, through a local mental health and counseling network, the Mental Health Initiative. This network is a collaboration between local and international NGOs, the Palestinian counseling center, the Palestinian Ministry of Health, and United Nations agencies. Only patients, aged more than one year, with a psychological disorder potentially related to a traumatic event were included in the MSF program. Patients with psychiatric chronic diseases or with only medical issues were excluded and referred to one of the two psychiatric hospitals of the Palestinian Ministry of health, for specific care.

For each patient included, a clinical evaluation was done by a psychologist or a psychiatrist, using a semi-structured interview based on criteria from the Diagnostic and Statistical Manual of Mental Disorders, Fourth Edition, Text Revision (DSM-IV-TR) [12]. Under the supervision of the senior MSF psychologist/psychiatrist, each evaluator was trained to complete a standardized and validated questionnaire. In case of psychopathological troubles, specific psychological care, consisting of individual, group, or dyad therapy [13,14], at home or in a consultation center, was offered. At the end of the treatment, or at the last visit, the condition of the patient was evaluated by a quantitative categorical analysis and classified in four categories (aggravated, unchanged, improved or cured). This classification was based on the number of symptoms and their severity in comparison with the initial evaluation.

Data were collected using a standardized questionnaire about sociodemographic characteristics (age, gender, geographic location (residence)), clinical features (patient-reported complaints, symptom type and severity, main and associated diagnostics identified per DSM-IV-TR), self-reported exposures to lifetime adverse events (frequency and type of traumatic event, family violence, chronic disease, history of psychiatric illness) and proposed psychological care (short-term psychotherapy type, length, and outcome). All patient information was entered anonymously, and oral informed consent was obtained before administration of the questionnaire.

Clinical and epidemiological data were entered into an EpiData database (EpiData, Odense, Denmark). Data for this study represent a secondary data source from which a descriptive analysis was performed. Therefore, no sample size or power calculation were performed since the analysis was conducted within the context of an existing cohort and the "sample" represented all patients enrolled in the program. Descriptive and statistical analyses were performed by age group (children ≤ 15 years old versus adults >15 years old), gender, and geographical area (Nablus district versus Gaza strip), using Epi Info version 6 (Centers for Disease Control and Prevention, Atlanta, GA, USA). Proportions or means were compared respectively by Chi-square test and independent samples t-test, with 5% significance level.

## Results

From January 2, 2005 to December 23, 2008, 1369 patients (773 from the Gaza strip and 596 from Nablus area) were identified and included in the MSF psychological care program (Table 1).

**Table 1:** Baseline characteristics of study population

	**Gaza strip** **n = 772**	**Nablus district** **n = 596**	**Total** **N = 1369**
Sex ratio (M/F)*	1.3 (443/329)	0.7 (244/352)	1.0 (687/681)
Median age (range)	15 years (14 months-83 years)	18 years (14 months-75 years)	16 years (14 months-83 years)
Place of residence			
Town	346 (45.0%)	203 (34.1%)	549 (40.2%)
Rural village	259 (33.7%)	198 (33.3%)	457 (33.5%)
Refugee camp	156 (20.3%)	194 (32.6%)	350 (25.6%)
Unknown	8 (1.0%)	0 (0%)	8 (0.6%)

* Sex missing for 1 patient

Among all patients, 50.2% were male, with a higher proportion of males in the Gaza patients (57.4%) than in the Nablus patients (40.9%) (p < 0.001). Median age at first consultation was 16 years (range: 14 months to 83 years); the Nablus patients were significantly older than the Gaza patients (mean: 24.3 years versus 19.5 years, p < 0.001). The geographic residence of patients was relatively evenly distributed between town (40.2%), rural village (33.5%), and refugee camp (25.6%).

At first consultation, the main complaint reported by patients was sadness (19.9%, 222/1117), followed by fear (19.4%, 217/1117). For 33.8% of patients (375/1110), the main clinical expression was distress or anxiety, and for over half of patients (58.3%, 456/1093), the severity of symptoms was considered moderate or mild. No other significant association was found between gender, severity and age or geographic residence (Nablus district versus Gaza strip).

Among the 1254 patients for whom clinical information was available, 291 patients (23.2%) met symptom criteria for PTSD; 217 (17.3%) for anxiety disorders other than PTSD and acute stress disorder; and 192 (15.3%) for depression (Table 2). PTSD was more frequently identified as the main diagnostic symptom in children ≤ 15 years old (25.8%) compared with adults, whereas depression was more frequently observed in adults (27.1%) than in children. For 348 patients (27.7%), at least two main psychological disorders were diagnosed, mainly other anxiety disorders associated with depression, PTSD or acute stress disorder. For 11.5% (144/1254) of the patients, no main diagnostic was identified.

**Table 2:** Main psychiatric disorders observed in the study population

	**Children ≤ 15 years** **n = 619**	**Adults** **n = 627**	Total* N = 1254
	**Number (%)**	**Number (%)**	**Number (%)**
Anxiety disorders**	113 (18.3)	103 (16.4)	217 (17.3)
Depression	21 (3.4)	170 (27.1)	192 (15.3)
Post-traumatic stress disorder (PTSD)	160 (25.8)	131 (20.9)	291 (23.2)
Acute stress disorder	40 (6.5)	52 (8.3)	94 (7.5)

* Age missing for 8 patients.

** Excludes acute stress disorder and PTSD.

For the majority of these patients, there was no history of chronic disease (92.9%) or prior psychological trouble (91.7%). However, 99.6% reported at least one traumatic event before the occurrence of psychological troubles. The three major traumatic events reported by patients were witnessing a murder or physical abuse (53.4%), property destruction or loss (32.7%), and killing of a close family member (31.5%) (Table 3). For 37.9% of patients (313/825), the traumatic event occurred more than 1 year before the first session. Intrafamily violence was reported for 264 of 1367 patients (19.3%).

**Table 3:** Patient-reported traumatic events

	**Children ≤ 15 years** **n = 650**	**Adults** **n = 667**	Total* N = 1325
	**Number (%)**	**Number (%)**	**Number (%)**
Witness to murder or physical abuse	337 (51.8)	359 (53.8)	702 (53.0)
Property destroyed or lost	238 (36.6)	192 (28.8)	433 (32.7)
Close family member killed	175 (26.9)	240 (36.0)	418 (31.5)
Received threats	145 (22.3)	218 (32.7)	367 (27.7)
Physical injury	101 (15.5)	220 (33.0)	322 (23.3)
Being forced to flee	155 (23.8)	109 (16.3)	266 (20.1)
Incarceration	67 (10.3)	130 (19.5)	198 (14.9)
Break-up of the nuclear family	62 (9.5)	83 (12.4)	146 (11.0)
Close family member died from illness	42 (6.5)	87 (13.0)	129 (9.7)
Sexual violence	6 (0.9)	16 (2.4)	22 (1.7)

* Age missing for 8 patients.

Among children ≤ 15 years old, factors strongly linked with PTSD included being witness to murder, being victim of sexual violence or physical abuse and receiving threats (p < 0.04). In adults, depression was significantly associated with being victim of sexual violence or physical abuse, receiving threats and having a close family member killed (p < 0.05).

Psychological care was conducted for all patients, mainly individual psychotherapy (65.1%), followed by family psychotherapy (22.6%) and dyad psychotherapy (12.3%). The proportion of group psychotherapy was higher for children ≤ 15 years old (50.1%) than for adults (20.0%; p < 0.001). Psychological care was conducted principally at the patient's home (67.7%). Among the 1339 patients for whom information on treatment was available, 30.6% required additional psychotropic medication (mainly Fluoxetine and Alprazolam). Median number of therapy sessions was 7.0 (range: 1-52). Median length of a full therapy was 12.0 weeks (range: 0-79), and one-third (33.0%) of patients required less than 8 weeks of therapy. The length of therapy was higher for children ≤ 15 years old than for adults (p = 0.05) (Table 4).

**Table 4:** Length and number of therapy sessions

	**Children ≤ 15 years** **n = 671**	**Adult** **n = 680**	Total* N = 1359
Number of sessions			
Mean [95%CI]	8.2 [7.8-8.6]	8.2 [7.7-8.6]	8.2 [7.9-8.5]
Median [range]	7.0 [1-52]	7.0 [1-50]	7.0 [1-52]

Number of weeks			
Mean [95%CI]	14.6 [13.8-15.5]	13.4 [12.6-14.3]	14.0 [13.4-14.6]
Median [range]	12.0 [0-79]	11.0 [0-71]	12.0 [0-79]

* Age missing for 8 patients

Among the 1122 patients for whom information was available, 886 (78.9%) had an improved condition at the last consultation. This proportion was higher for children ≤ 15 years old (82.8%) than for adults (75.3%; p = 0.001), and for the Gaza patients (83.3%) than for the Nablus patients (75.1%, p < 0.001). No other significant association was found between greater improvement at the last consultation and gender or intake of medications. For patients whose condition at the last session remained unchanged or aggravated, the main persistent symptoms were sadness (14.0%) and aggressive behavior (12.7%).

## Discussion

We describe the occurrence of severe psychological disorders in Palestinian populations from 2005 to 2008, in the Gaza strip and West Bank exposed to repeated traumatic experiences. Based on the DSM-IV-TR, the three main psychological disorders diagnosed during this 42-month period were PTSD, depression, and anxiety disorders (excluding PTSD and acute stress disorder). These findings were similar to those described in previous studies [8,15,16].

In our study, differences in the expressed pathology were observed between adults and children ≤ 15 years of age. Whereas depression was observed in one-third of adults, in children ≤ 15 years old PTSD was the main symptom. Several studies concentrating on Palestinian children or adolescents have found that they were at high risk of PTSD [8,15,17]. In our study, no difference between children ≤ 12 years old and adolescents aged 13-18 years was observed (*data not shown*).

An association between self-reported exposure to specific traumatic events and PTSD in children ≤ 15 years was found in our study population. However, it is difficult to separate the effect of war trauma from that of potential confounding factors such as pre- and post-migration stress, family separation, socioeconomic adversities, and acculturation difficulties [18]. Moreover, no details regarding the specific nature (exposure to actual physical abuse e.g. family violence, wife beating, child abuse), timing (in particular the timing of onset of psychological disorders relative to major military or political events), and severity of the traumatic events reported were collected in our questionnaire. Another limitation is that we are not certain about the accuracy of the self-reported data. Also, there are likely differences in respondent recall of trauma and their lifetime sequelae.

These findings should be examined in light of the characteristics of our study population. We focused solely on patients recruited through home visits in limited areas. Each of the Occupied Palestinian Territories has a unique set of circumstances, and generalization is difficult. Our results are not able to provide sufficient information to show potential differences between the characteristics of the patients living in Gaza strip and the ones living in Nablus district, where experiences may differ substantially under the Hamas and Palestine Liberation Organization administration. Nevertheless, even if our studied population was not representative of the Occupied Palestinian Territories, our results indicate the extent of psychological disorders in selected catchment areas affected by the conflict and highlight a population who required early psychological support.

Despite difficulties implementing a psychological program in this context, our study reported that nearly 80% of the patients had improved symptoms. Less than a third of patients were prescribed medication, pointing to the potential effectiveness of psychological support by non-medical interventions. Also, though therapy was longer in children than in adults, children recovered more frequently than adults. This finding reinforces the notion that short-term psychotherapy could be an effective treatment in specific psychiatric disorders, especially children [11].

Further research, using prospective and/or longitudinal design, is needed to address the long-term impact of short-term therapy on the psychological well-being of a vulnerable population living in a context of conflict and violence.

## Competing interests

The authors declare that they have no competing interests.

## Authors' contributions

*Conception and design or acquisition of data*: TB, GC, VG MRM, YM

*Analysis and interpretation of the data*: GC, EE, VG, RFG, MRM.

*Drafting of the manuscript*: EE.

*Critical revision of the manuscript for important intellectual content*: TB, GC, EE, VG, RFG, MRM, YM, OY.

*Final approval of the version to be published*: TB, GC, EE, VG, RFG, MRM, YM, OY.
